# A Breast Cancer Smartphone App to Navigate the Breast Cancer Journey: Mixed Methods Study

**DOI:** 10.2196/28668

**Published:** 2021-05-10

**Authors:** Serena Petrocchi, Chiara Filipponi, Giacomo Montagna, Marta Bonollo, Olivia Pagani, Francesco Meani

**Affiliations:** 1 Institute of Communication and Health Università della Svizzera Italiana Lugano Switzerland; 2 Applied Research Division for Cognitive and Psychological Science European Institute of Oncology IRCCS Milan Italy; 3 Breast Surgery Service Memorial Sloan Kettering Cancer Center New York, NY United States; 4 Gynecology and Obstetrics Service Centromedico Mendrisio Switzerland; 5 Swiss Group for Clinical Cancer Research Geneva University Hospitals Geneva Switzerland; 6 Department of Obstetrics and Gynecology Centro di Senologia della Svizzera Italiana Ente Ospedaliero Cantonale Viganello Switzerland

**Keywords:** breast cancer, decision-making process, breast cancer patient, smartphone app, empowerment, breast cancer journey, mobile app

## Abstract

**Background:**

Several mobile apps have been designed for patients with a diagnosis of cancer. Unfortunately, despite the promising potential and impressive spread, their effectiveness often remains unclear. Most mobile apps are developed without any medical professional involvement and quality evidence-based assessment. Furthermore, they are often implemented in clinical care before any research is performed to confirm usability, appreciation, and clinical benefits for patients.

**Objective:**

We aimed to develop a new smartphone app (Centro di Senologia della Svizzera Italiana [CSSI]) specifically designed by breast care specialists and patients together to help breast cancer patients better understand and organize their journey through the diagnosis and treatment of cancer. We describe the development of the app and present assessments to evaluate its feasibility, usefulness, and capability to improve patient empowerment.

**Methods:**

A mixed method study with brief longitudinal quantitative data collection and subsequent qualitative semistructured interviews was designed. Twenty breast cancer patients participated in the study (mean age 51 years, SD 10 years). The usability of the app, the user experience, and empowerment were measured after 1 month. The semistructured interviews measured the utility of the app and the necessary improvements.

**Results:**

The app received good responses from the patients in terms of positive perception of the purpose of the app (7/20, 35%), organizing the cure path and being aware of the steps in cancer management (5/20, 25%), facilitating doctor-patient communication (4/20, 20%), and having detailed information about the resources offered by the hospital (2/20, 10%). Correlation and regression analyses showed that user experience increased the level of empowerment of patients (B=0.31, 95% CI 0.22-0.69; *P*=.009). The interviews suggested the need to constantly keep the app updated and to synchronize it with the hospital’s electronic agenda, and carefully selecting the best time to offer the tool to final users was considered crucial.

**Conclusions:**

Despite the very small number of participants in this study, the findings demonstrate the potential of the app and support a fully powered trial to evaluate the empowering effect of the mobile health app. More data will be gathered with an improved version of the app in the second phase involving a larger study sample.

## Introduction

### Background

The time of cancer diagnosis is uncertain and worrisome. Several studies on cancer patients have shown a strong desire for information and guidance, and these needs are often unmet [1-3]. A patient binder has been introduced in many breast units to provide patients with clear and easy to understand information, as well as to organize and manage appointments, treatment schedules, medical documents, and contact details. It has been proven that such a tool could help to reduce psychological distress and improve adaptation to cancer [4,5]. In the current technological era, the old concept of the patient binder has been improved and modernized. Mobile phone apps have been designed to operate as an electronic version of the patient binder. This modern way of conveying information is believed to be even more efficacious in enhancing communication between the health care institution and patients [6,7]. Electronic applications are often programmed to include the same sections of the old binders, with the advantage of unique portability and adaptability [8].

The use of mobile apps has grown exponentially in the last few years, particularly for cancer patients [9,10], as they carry the potential to provide health-pertinent information, services, and even health care interventions in a cost-effective way. Current estimates report more than 40,000 health-related applications available on the market, often freely delivered [11]. In particular, in the field of oncology, health apps have been employed to promote prevention, promote early detection, manage cancer care, and support survivorship [12,13]. Unfortunately, despite the promising potential and impressive spread, their effectiveness often remains unclear. Mobile apps are, most of the time, developed without any quality assessment procedure (evidence based) and medical professional involvement. Furthermore, they are often implemented in clinical care before any research is done to confirm usability, appreciation, and clinical benefits for patients [11].

Two recent systematic reviews on research-tested apps for breast cancer [14,15] showed promising results from studies on the inclusion of health apps in breast cancer care and called for caution before implementing these apps in clinical practice, as the final effects on users may be unpredictable [16,17] or not disease focused [15]. Therefore, they need to be extensively and scientifically tested before making them available to the public [14,15,18].

From this standpoint, the Centro di Senologia della Svizzera Italiana (CSSI) endeavored in designing and developing (proof of concept) a new smartphone app dedicated to breast cancer patients and based on direct observation of patients’ needs in everyday practice. Patients actively participated in meetings with medical staff and software developers during the whole process to develop the prototype and fine tune the tool according to their needs before this pilot test run.

The aim of the app is to help patients better understand and organize their journey through the diagnosis and treatment of cancer. In other words, it aims to develop self-management skills and to empower them in the decision-making process for treatment plans. Through psychological empowerment, individuals gain control over their lives [19] and develop cognitive abilities to deal with difficult situations and behavioral tendency to take actions [20]. Psychological empowerment is one of the most important constructs for an individual’s well-being. Therefore, the app was developed to give women the ability to promote active participation in their health care, improve their sense of control, and improve the relationship and communication with their attending physicians. The app was designed to allow patients to be able to quickly access reliable information related to breast cancer and the treatment process. It offers the possibility to carry important selected medical files for second opinions or for sharing with caregivers/partners, to have a private electronic agenda available for appointments and medical checks, which can also be remotely updated by the breast unit staff, and to allow the patient to write down questions and notes on an electronic scratchpad to recall at the time of the visit. The app also provides a telephone directory with useful contact details to reach the treating team and delivers news regarding activities, conferences organized by the treating institute, and other relevant information for the patients.

Access to the content of the app is made secure by encryption and by login through a two-factor identification system. Before implementation on a large scale in clinical practice, we designed a pilot test for usability and qualitative evaluation of the app prototype by a small group of breast cancer patients to build an evidence-based foundation for its use. The test run involved patients who received a breast cancer diagnosis and focused on the following three specific areas of investigation: (1) the perceived usefulness of the app, (2) the possible effect that the use of the app may have on women empowerment, and (3) the improvements or implementations proposed by the patients themselves. The goal was to show a development model for an app dedicated to breast cancer patients, but potentially extendable to other cancer patients.

### App Layout and Features

The CSSI app is organized to provide convenient access to information and selected relevant documents according to the different phases of the cancer journey. The home page offers a central large “news banner” through which the breast unit can keep patients and users updated on the activities and services that are offered. Besides, several different widgets help navigate through other sections of the app. The links section provides links to reliable breast cancer websites to access several quality information sources (prevention, detection, diagnosis, treatment, survivorship, support groups, services, etc) carefully selected and periodically updated by our team. The contacts section provides suitable information to reach the CSSI health care team members and facilities. The patient’s calendar helps to keep all patient appointments organized and secured in one place and also allows patients to put reminders and keep track of what has been arranged for them. The patient’s documents section allows patients to store relevant clinical reports (radiology reports, blood test reports, histology data, and descriptions of surgical interventions). For patients, it is essential to have easy access to their relevant clinical information in order to be able to share them, for example, with other doctors in case of second opinions. The note pad appears as a loose-leaf paper and is suitable for notetaking and jotting down questions patients want to ask their health care team, as well as recording the answers, and for easily finding notes when needed.

### Aims

The first aim of this study was to test the app usability for the very first time and collect information about the patients’ perceived usefulness of the app. The second aim concerned the consideration of the possible positive effects that the use of the app may have on patient empowerment. Finally, the third area of interest in this study was the analysis of improvements or implementations as proposed by the patients.

## Methods

### Procedure

Between February 2017 and August 2018, encouraged and supported by an active group of our young patients (Anna dai Capelli Corti) [21], we developed at the Breast Unit of Italian Switzerland (CSSI) a new smartphone app specifically dedicated to breast cancer patients. The development of the app was based on direct observation of our patients’ needs in everyday practice. To help us design it, patients actively participated in meetings with medical staff and software developers and provided feedback on the evaluation of the prototype in order to increase its readability, ease of use, and adherence to patient needs.

Following the prototype set up, between August 2018 and February 2020, a total of 20 patients, treated at the CSSI, were invited to take part in a test run of the new information technology (IT) tool explicitly designed for aiding breast cancer patients with their journey through cancer treatments. The project protocol was drawn up following the indications of the Declaration of Helsinki on research involving human subjects, and the Ethical Committee of the Università della Svizzera Italiana approved the research. The participants did not receive any compensation for taking part in the project.

The patients were invited by their attending physician to participate in this research project. Inclusion criteria were age over 18 years, diagnosis of nonmetastatic operable breast cancer, and ownership of a smartphone. Adherence to this pilot project was proposed shortly after surgery at the time of the first postoperative check (T0). The breast specialist was in charge of explaining the project and obtaining informed consent with a signature from the patient. The secretarial staff briefly illustrated the structure and functions of the app and helped the patients to download it and obtain the login credentials.

Patients were asked to use the CSSI app for a minimum of 1 month. Afterward, while under adjuvant treatments or follow-up, the patients were reconvened to complete a paper questionnaire and reply to a short interview (T1). The questionnaire collected sociodemographic information (age, degree of education, and marital status), information relating to the use of the app, a subjective assessment of the characteristics of the app, and judgment regarding the personal perception of the support derived from the use of the app in managing one’s care path. The short semistructured interview (lasting about 5-10 minutes) aimed at investigating the usefulness of the app, as perceived by patients, and collecting suggestions for improvements to be made to the tool or its management.

### Participants

Twenty patients participated in this pilot project (mean age 51 years, SD 10 years), and all of them had an education level exceeding mandatory schooling and a partner. Table 1 presents detailed information about the sample’s characteristics.

**Table 1:** Characteristics of the participants (N=20).

Characteristic		Value, mean (SD) or n (%)
Age (years), mean (SD)		51 (10)
**Education, n (%)**		
	Apprenticeship/high school diploma	12 (60%)
	University bachelor’s degree	5 (25%)
	University master’s degree	3 (15%)
**Marital status, n (%)**		
	Single	3 (15%)
	Married or civil partnership	13 (65%)
	Separated or divorced	3 (15%)

### Measurement Tools

#### Questionnaires

##### Usage Habits

A scale consisting of six items taken from the existing Mobile App Rating Scale (MARS) [22] was applied to investigate the familiarity of the participants to the use of electronic devices (in general, and smartphones in particular) in everyday life. Specifically, questions asked the frequency of smartphone use (from 1 [several times a day] to 4 [never]), the use of other apps in addition to that provided by the hospital (yes/no), the types of apps used (gaming and entertainment apps, information apps, social apps, or any other), and the frequency of use (from 1 [several times a day] to 3 [a few times during the week]).

##### Usability of the App

Thirteen items were selected from the MARS to investigate the patients’ subjective assessment of the app in terms of its goal, interest of usage, appropriateness of the content, easiness of functioning, reliability of the information provided, design, learning process needed to use the app, trustfulness of the source, intention of using the app in the future, and overall app evaluation. The first item (ie, assessment of the app in terms of its goal) presented a multiple-choice response with six possible options (ie, increase well-being, decrease negative emotions, organize the care process, inform about the services offered by the hospital, increase awareness, and control over the care process and simplify the relationship with the doctor). All the other items had response options ranging from 1 to 5 (with labels according to the item). Items 7, 8, and 9, which all evaluated the external graphic aspect of the app, were averaged, and the final score showed acceptable reliability and a moderate internal consistency (α=.58, rs>0.29). All other items were considered individually.

##### User Experience

The scale is made up of the following seven items developed ad-hoc for this study: “I think I would like to use this app frequently,” “I found it complicated to use,” “I think I need the support of a person who is already able to use it,” “I think the features of the app are well integrated,” “I found inconsistencies between the various app features,” “I think most people could learn to use the app easily,” and “I used the app with confidence.” Response options ranged from 1 (completely disagree) to 5 (completely agree) relating to the statements. A general indicator of user experience was created by averaging the individual items. The internal consistency calculated on the seven items was low (α=.57, rs>0.18), and Cronbach alpha suggested eliminating three items (“I found it complicated to use,” “I think most people could learn to use the app easily,” and “I used the app with confidence”). On recalculating the internal consistency for the remaining four items, alpha reached acceptable levels (α=.64, rs>0.33).

##### Empowerment

The scale consists of nine items selected from the Empowerment Scale [23]. An assessment was performed on a scale from 1 (in full disagreement) to 7 (completely agree) relating to a list of statements concerning the breast cancer treatment experience after app use. The total measure of empowerment was calculated as the average of the responses to the individual items and showed good internal consistency (α=.97, rs>0.81).

#### Interview

The semistructured interview was focused on the following two specific areas of investigation: the perceived utility of the app and the possible areas of improvement, with very general and open questions to reduce possible bias and unreliability of the answers.

## Results

### Smartphone Usage Habits

Most participants declared using the app on their smartphone several times per week (18/20, 90%). Our patient population was composed of women accustomed to mobile apps in general. The majority of them (18/20, 90%) declared keeping and using other apps on their smartphone, for example, information apps (eg, broadcast), entertainment apps (eg, games), and social apps to keep in contact with others (eg, WhatsApp Messenger). They also declared that they used those apps several times in a day (16/20, 80%).

### App Usability

According to the results of the questionnaire, in terms of perception of the purpose of the app, 35% (7/20) of women declared that the tool helped them to organize the cure path and to be more aware of what is the next step in management (5/20, 25%). Another 20% (4/20) declared that the app facilitated communication with the doctor, and 10% (2/20) said that they have a clear idea about the health offered by the hospital owing to the app. A small percentage (1/20, 5%) declared that the app helped to manage negative emotions.

Table 2 shows descriptive statistics regarding the other MARS items. The maximum possible value was 5. As can be seen in the table, the mean values are quite high (>3.46), except for the values of the questions regarding trustfulness and the possibility to use the app even in the future.

**Table 2:** Descriptive statistics of the Mobile App Rating Scale.

Item	Score		
	Range	Mean	SD
Interest of use	2-5	4.00	0.81
Appropriateness of the contents	3-4	3.61	0.50
Easiness of functioning	2-4	3.46	0.66
Accuracy of the information	4-5	4.33	0.49
Learning process	3-5	4.46	0.66
App design	3-5	3.77	0.43
App recommendation	1-5	3.75	1.20
Trustfulness of the source	1-3	2.16	1.02
Use of the app in the future	2-3	2.41	0.51
General evaluation	2-5	3.58	0.90

### Relations Among Variables

Table 3 shows the correlations among the variables. Sociodemographic variables were negatively correlated with the MARS. Specifically, older patients put higher efforts in understanding how to use the app. Moreover, a higher level of education was associated with a lower positive general evaluation of the app. Women who perceived a higher interest in using the app declared a higher general positive evaluation, in addition to those who would recommend the app and who evaluated the content as appropriate and the design as high quality. Patients who evaluated the content as appropriate revealed a higher probability of using the app in the future and higher empowerment scores. The level of patient empowerment after using the app for a month correlated with the evaluation of the appropriateness of the content, the design of the app, the recommendations found in the app, the possibility of using the app in the future, and the general evaluation of user experience.

The regression analysis findings in Table 4 demonstrate that empowerment increased when women perceived the content of the app as appropriate, appreciated the quality of the design, and were satisfied with the general user experience (step 2). Age was also a significant and positive predictor of empowerment (step 2).

**Table 3:** Correlations among the variables.

Variable		Age	Educational level	Interest of use	Appropriateness of the contents	Easiness of functioning	Accuracy of the information	Learning process	App design	App recommendation	Trustfulness of the source	Use of the app in the future	General user evaluation	User experience	Empowerment
**Age**															
	*r*	1	0.29	0.10	−0.10	−0.07	−0.03	−0.68	0.23	0.11	−0.32	0.15	−0.09	0.17	0.14
	*P* value	—^a^	.37	.75	.75	.82	.93	.01	.47	.72	.31	.65	.78	.60	.65
**Educational level**															
	*r*	0.29	1	0.09	−0.14	−0.42	0.28	−0.18	0.39	−0.34	0.14	0.00	−0.72	−0.16	−0.11
	*P* value	.37	—	.77	.65	.18	.40	.58	.21	.28	.67	.99	.009	.61	.74
**Interest of use**															
	*r*	0.10	0.09	1	0.27	−0.25	0.12	0.29	0.56	0.58	0.03	0.58	0.33	0.19	0.56
	*P* value	.75	.77	—	.39	.42	.72	.37	.06	.049	.93	.034	.29	.55	.06
**Appropriateness of the contents**															
	*r*	−0.10	−0.14	0.27	1	−0.06	−0.39	0.20	0.21	0.45	−0.24	0.60	0.29	0.16	0.62
	*P* value	.75	.65	.39	—	.86	.24	.52	.51	.14	.45	.04	.37	.62	.03
**Easiness of functioning**															
	*r*	−0.07	−0.42	−0.25	−0.06	1	−0.07	−0.14	−0.16	−0.07	−0.52	0.22	0.27	−0.27	−0.16
	*P* value	.82	.18	.42	.86	—	.84	.67	.61	.82	.09	.50	.39	.39	.63
**Accuracy of the information**															
	*r*	−0.03	0.28	0.12	−0.39	−0.07	1	−0.36	0.17	−0.07	0.46	−0.04	−0.39	0.50	0.13
	*P* value	.93	.40	.72	.24	.84	—	.27	.62	.84	.15	.91	.24	.12	.71
**Learning process**															
	*r*	−0.68	−0.18	0.29	0.20	−0.14	−0.36	1	0.24	0.06	0.22	0.08	0.46	−0.16	0.22
	*P* value	.01	.58	.37	.52	.67	.27	—	.45	.85	.49	.80	.13	.62	.49
**App design**															
	*r*	0.23	0.39	0.56	0.21	−0.16	0.17	0.24	1	0.48	0.00	0.63	0.11	0.21	0.74
	*P* value	.47	.21	.06	.51	.61	.62	.45	—	.11	.99	.03	.74	.52	.006
**App recommendation**															
	*r*	0.11	−0.34	0.58	0.45	−0.07	−0.07	0.06	0.48	1	−0.18	0.66	0.43	0.48	0.75
	*P* value	.72	.28	.049	.14	.82	.84	.85	.11	—	.58	.02	.16	.12	.005
**Trustfulness of the source**															
	*r*	−0.32	0.14	0.03	−0.24	−0.52	0.46	0.22	0.00	−0.18	1	−0.31	−0.03	0.36	0.02
	*P* value	.31	.67	.93	.45	.09	.15	.49	.99	.58	—	.32	.93	.26	.94
**Use of the app in the future**															
	*r*	0.15	0.00	0.58	0.60	0.22	−0.04	0.08	0.63	0.66	−0.31	1	0.38	0.36	0.75
	*P* value	.65	.99	<.05	.04	.50	.91	.80	.03	.02	.32	—	.22	.26	.006
**General user evaluation**															
	*r*	−0.09	−0.72	0.33	0.29	0.27	−0.39	0.46	0.11	0.43	−0.03	0.38	1	0.25	0.47
	*P* value	.78	.009	.29	.37	.39	.24	.13	.74	.16	.93	.22	—	.44	.13
**User experience**															
	*r*	0.17	−0.16	0.19	0.16	−0.27	0.50	−0.16	0.21	0.48	0.36	0.36	0.25	1	0.59
	*P* value	.60	.61	.55	.62	.39	.12	.62	.52	.12	.26	.26	.44	—	.043
**Empowerment**															
	*r*	0.14	−0.11	0.56	0.62	−0.16	0.13	0.22	0.74	0.75	0.02	0.75	0.47	0.59	1
	*P* value	.65	.74	.06	.03	.63	.71	.49	.006	.005	.94	.006	.13	.043	—

^a^Not applicable.

**Table 4:** Regression analysis.

Variable		Empowerment	
		Β (95% CI) or %	*P* value
**Step 1: Sociodemographic**			
	Civil status (0=no partner)	0.09 (−1.37 to 1.73)	.79
	Age	0.34 (−0.04 to 0.12)	.33
	Education (0=low)	−0.12 (−1.35 to 1.01)	.74
	R^2^ (%)	13%	N/A^a^
**Step 2: Experience**			
	Civil status (0=no partner)	−0.16 (−0.64 to 0.03)	.06
	Age	0.13 (0.001 to 0.03)	.04
	Education (0=low)	−0.15 (−0.55 to 0.12)	.13
	Appropriateness of the contents	0.44 (0.63 to 1.39)	.003
	App design	0.61 (0.81 to 2.34)	.007
	App recommendation	0.18 (−0.09 to 0.43)	.12
	Use of the app in the future	−0.02 (−0.56 to 0.44)	.71
	User experience	0.31 (0.22 to 0.69)	.009
	R^2^ (%)	79%	N/A

^a^N/A: not applicable.

### Perceived Usefulness and Suggested Improvements

At the end of the test period and after filling up the questionnaire, the patients were asked to give their opinions on the app through a semistructured interview.

The first area investigated the perceived usefulness and the reasons for their perception. Overall, 55% (11/20) of the patients declared that the app was in general useful. Some examples of the patient responses are as follows:

Yes, very useful for having all the appointments in the same application and for accessing information links.

age 55 years

Yes, to keep track of appointments.

age 58 years

It was particularly useful for finding reliable and verified information.

age 54 years

The other patients (9/20, 45%) declared that the app was partially useful. Some examples of responses are as follows:

Partially useful, I find the part of the contact details very useful especially when you are worried, but I find the part relating to the reports lacking because they are loaded only at the request of the patient.

age 60 years

There are aspects that can help, but at the same time some limits: I would increase communication between the various departments/health workers involved in the App management.

age 55 years

No, since the agenda was not updated, I preferred to use my own (agenda).

age 55 years

When focusing on communication, the majority of patients (15/20, 75%) did not believe that the app had influenced their relationship with the referring physician. Only a minority (4/20, 20%) of patients reported that using the app helped them to create a more direct channel of communication with the doctor, while the remaining 5% (1/20) of patients replied that they did not have a clear opinion on this aspect.

As for cancer management, the majority of patients (13/20, 65%) declared not considering the app useful as a self-management tool. Some of the reasons reported by the patients to explain this point were that the app reminded them of being sick or that they felt capable of managing cancer and the related treatment on their own. Interestingly, however, more than half of the patients (11/20, 55%) affirmed that the app increased the knowledge they had about their clinical condition and thus the awareness of their own path of clinical care.

The second area of investigation focused on the proposed improvements. All patients provided several suggestions on different aspects.

First, regarding treatment modalities, patients suggested increasing the number of links to reliable websites, with a focus on surgical treatments, pre- and postsurgical images, and news on newly developed technologies. Information on remedies to manage the side effects of cancer treatments (eg, use of laser during menopause) was frequently requested. Second, patients suggested features for self-management, with inclusion of an alarm as a reminder for medical appointments or a notification when the hospital cancels an appointment. Third, patients suggested including information regarding activities and/or resources (conferences within the hospital or other health institutions, or the existence of patient advocacy groups).

A special area of improvement focused on the doctor-patient relationship with the inclusion of a section dedicated to bidirectional communication between health care providers and patients. Interestingly, another area of improvement concerned the inclusion of a section dedicated to the costs of medical treatment. Finally, 12 patients spontaneously suggested delivering the app at a different time, preferentially before treatment, to increase its usefulness as a source of reliable information.

## Discussion

### General Opinions on the App’s Usefulness

This work describes a structured process involving end-users to test the efficacy and perceived usefulness of a new mobile app dedicated to breast cancer patients. We also investigated possible areas of improvement for further development as proposed by the patients themselves. The aim of the app was to help patients better understand and organize their journey through the diagnosis and treatment of cancer, that is, to develop their self-management skills, empowerment, and sense of control. Overall, our patients perceived the app as easy to learn and use, accurate, and appropriate. They stated that they would use the app in the future and rated it almost 4 out of 5 points. Moreover, the app increased patient empowerment.

### Association of the App’s Perceived Usefulness, Sociodemographic Characteristics, and Empowerment

We found significant relationships between variables. As expected, older patients put higher efforts in understanding how to use the app compared with younger patients. This is an expected result given the use of technologies is more common among young people. Future development of the app should take into account the age influence, mainly because breast cancer risk increases with age, peaking above 50 years [24,25].

The educational level was negatively correlated with general users’ evaluation, that is, a higher level of education was associated with a lower positive general evaluation of the app. These results might be linked to the fact that the educational level is associated with literacy and health literacy [26,27]. It may be that participants with high educational levels have high literacy and therefore high knowledge and competence with the use of new technologies. As expected, there were correlations between the items of the MARS, indicating a general and overall consistency of the app rating. Women who perceived a higher interest in using the app declared a higher general positive evaluation, in addition to those who recommended the app and who evaluated the content as appropriate and the design as high quality.

However, the most interesting result was regarding the level of patient empowerment, which increased when women perceived the content of the app as appropriate, perceived the high quality of the design, and were satisfied with the general user experience of the app. This result represents the first demonstration of the perceived usefulness of the app by breast cancer patients, which was a fundamental aim of the research. Therefore, apart from a generally positive evaluation of the app, it seems that its use enhanced the sense of control over cancer and the general empowerment of women owing to the potential of technologies to switch from a paternalistic to a collaborative relationship between patients and physicians [28,29].

### Suggested Improvements

From the interviews, it was found that our app, as with other mobile health (mHealth) tools, will require constant revision and updates. Nowadays, the information flow on the internet and social media is constantly in dynamic change. Future development of the app should take into account that a static app functioning as a sort of agenda without being connected to the hospital agenda, for example, or without timely updates is not useful.

There appeared to be inconsistency in the results concerning the perceived usefulness of the app. While 65% of patients declared a certain level of disappointment with the app as a self-management tool, more than half (55%) of the patients declared that it helped to increase knowledge about their clinical condition and awareness of their own path of clinical care. This might be a demonstration of the discrepancy between the effect delivered by the use of the tool and the awareness of real advantages that are not fully perceived as such. In this sense, our preliminary results seem to suggest that use of the app holds the potential to improve the sense of control over cancer and the general empowerment of women [28], which are both related to positive health outcomes [30-32].

Another very important aspect arising from the interviews is that the vast majority of patients perceived receiving the app after surgery as utterly untimely. They stressed that the app was not available as a source of reliable information when they needed it the most (before treatment). Such feedback is practice changing and will be taken into account in the next validation phase and finally in routine clinical practice. This is, in our opinion, a clear example of the utility of patient involvement in the development and implementation process of mHealth tools.

### Limitations

This study has several limitations. First, the sample showed selection bias, and its size was quite small. Our sample included a small number of breast cancer patients selected via word of mouth. These patients used the smartphone quite frequently and were accustomed to using other apps. In this sense, the sample involved women with an adequate level of literacy regarding the use of electronic tools such as the smartphone and apps. Women with a lower literacy level were underrepresented. Future research should test the app with older patients and with a larger sample size. It might also be that women who agreed to participate were the most involved in their care path, and this may represent a selection bias. Second, this study did not consider a control group of women with no access to the app for comparison. Future research should include two groups of women and measure the baseline empowerment at the time of enrollment in the research. Third, in this pilot study, the semistructured interview was conducted by the treating physician. This might have consciously or unconsciously altered the way answers were given or interpreted (observation and interviewer bias). Finally, the sample considered here included patients with breast cancer. Future research should test the app with heterogeneous groups of patients having different diagnoses and types of treatments.

### Conclusions

The values of this work and the CSSI app lie in the involvement of health and communication professionals in the design and implementation processes. Of equal value is the assessment of the quality and usability of the contents performed through the involvement of patients in a feedback process guided by scientifically validated questionnaires. In this way, our mHealth tool differs from the vast majority of other health apps on the market, which are often produced without the involvement of health care professionals and patients, devoid of any scientific basis, and not subjected to any quality assessment. We learned that the IT tool has maximum utility and obtains maximum consent when managed correctly by staff and when its features and use are clearly explained to final users. It requires constant application by managers to guarantee effective functioning and the continuous updating of content. Patient feedback also underlined the importance of the timing and delivery methods of the app. It takes time to explain how it works, even if the tool is simple. Our preliminary data seem to suggest that the best time to offer it is before surgery, but not when communicating the diagnosis. This aspect will be further investigated in the second phase of our work. Among the various features, unlike what was expected at the time of the app design, those that attracted the most attention of the patients were the electronic appointment calendar and the storage area for clinical reports.

Findings from this pilot study demonstrate the potential of the app and its validation protocol, and support a fully powered trial to evaluate the empowering effect of the mHealth app. More data will be gathered with an improved version of the app in a second phase involving a larger study population. The next step will be to extend the use to a greater number of patients and follow our patients’ suggestions. We will ensure a more proactive attitude by the team responsible for the management of the IT tool and the interaction with users. We will implement changes to the software suggested through feedback, and at that point, a new assessment will be performed for quality, appreciation, and usefulness in terms of patient empowerment of the sense of control and self-management.

## Abbreviations

CSSI: Centro di Senologia della Svizzera Italiana
IT: information technology
MARS: Mobile App Rating Scale
mHealth: mobile health
